# LIVE DISCHARGE FROM HOSPICE AS A CRITICAL CARE TRANSITION: MEASUREMENT, EXPERIENCES, AND SOLUTIONS

**DOI:** 10.1093/geroni/igae098.1795

**Published:** 2024-12-31

**Authors:** Cara Wallace, Lauren Hunt

**Affiliations:** Chair; Discussant

## Abstract

Hospice care has been shown to improve end-of-life outcomes for adults with chronic illness, yet with eligibility limited to a six-month prognosis, the hospice system is not structured to meet longer-term needs. Many adult patients stabilize, or have a change in terminal prognosis, leading to a ‘live discharge.’ In 2021, 17.2% of all Medicare discharges were patients discharged alive, with 6.3% of these due to decertification (patient stabilization). This symposium examines live discharge as a critical care transition, including potential measures of quality and continuity, how hospice care coordination or caregivers’ social contexts impact experiences of the transition, and a potential intervention to improve the overall process. The first presentation considers utility of the Care Transition Measure (CTM-15) and the Patient-Perceived Continuity of Care from Multiple Clinicians Measure in live discharge from hospice, highlighting the positive and statistically significant relationship between relational continuity—how well a patient feels known and understood by their primary hospice clinician and overall team—and the quality of the care transition. Next, using scores from the CTM-15 to classify participants’ experiences as “positive” or “negative,” presenters will provide information about the important role of hospice care coordination in the overall experience of the transition off hospice. Third, presenters will showcase the social context among ADRD caregivers, including the way support systems help caregivers during and following a discharge. Last, presenters will demonstrate the development and preliminary usage of a Live Discharge Protocol that hospices can use for a standardized approach to improving this care transition.

